# Gut Dysbiosis and Fecal Microbiota Transplantation in Autoimmune Diseases

**DOI:** 10.3390/ijms231810729

**Published:** 2022-09-14

**Authors:** Paulina Belvoncikova, Martin Maronek, Roman Gardlik

**Affiliations:** Institute of Molecular Biomedicine, Faculty of Medicine, Comenius University, 811 08 Bratislava, Slovakia

**Keywords:** dysbiosis, fecal microbiota transplantation, rheumatoid arthritis, multiple sclerosis, type 1 diabetes, systemic lupus erythematosus, celiac disease, Sjögren’s syndrome, Hashimoto’s disease, Graves’ disease

## Abstract

Gut microbiota dysbiosis has recently been reported in a number of clinical states, including neurological, psychiatric, cardiovascular, metabolic and autoimmune disorders. Yet, it is not completely understood how colonizing microorganisms are implicated in their pathophysiology and molecular pathways. There are a number of suggested mechanisms of how gut microbiota dysbiosis triggers or sustains extraintestinal diseases; however, none of these have been widely accepted as part of the disease pathogenesis. Recent studies have proposed that gut microbiota and its metabolites could play a pivotal role in the modulation of immune system responses and the development of autoimmunity in diseases such as rheumatoid arthritis, multiple sclerosis or type 1 diabetes. Fecal microbiota transplantation (FMT) is a valuable tool for uncovering the role of gut microbiota in the pathological processes. This review aims to summarize the current knowledge about gut microbiota dysbiosis and the potential of FMT in studying the pathogeneses and therapies of autoimmune diseases. Herein, we discuss the extraintestinal autoimmune pathologies with at least one published or ongoing FMT study in human or animal models.

## 1. Gut Microbiota and Fecal Microbiota Transplantation

The role of the gut microbiome in the context of immunity has been a very popular topic in the past years. Various studies have suggested that gut microbiota could have an impact on the onset and pathogenesis of numerous autoimmune diseases. Whereas the majority of studies have focused on intestinal disorders, changes in the gut microbiota composition have also been described in extraintestinal ones, such as rheumatoid arthritis, multiple sclerosis, type I diabetes, systemic lupus erythematosus and celiac disease [1,2,3,4,5,6,7,8,9]. Despite the diverse methodological procedures (amplicon vs. whole-genome sequencing, operational taxonomic unit vs. amplicon sequence variant approach), differences in study participants (age, gender, nationality, previous treatment) and variability of microbiome compositions oftentimes resulting in compositional inconsistencies, dysbiosis has been widely presented in all of these. It is inevitable that some changes occur, but it is not fully understood whether gut microbiota dysbiosis is an active trigger affecting the onset or is the consequence of autoimmune inflammatory processes. In order to target gut microbiota as a potential modulator in the pathogenesis of autoimmune disorders, there are several interventions available, such as the administration of antibiotics, probiotics, prebiotics, synbiotics or fecal microbiota transplantation (FMT). FMT refers to a method by which donor gut microbiota is transferred into the digestive tract of the recipient, aiming to restore gut microbial imbalance towards eubiosis. During the procedure, the fecal matter of a healthy donor is delivered via either a nasogastric tube, colonoscope or capsule method [10]. The studies have proposed that FMT represents a safe procedure [11,12,13]. The very first record on FMT dates to the 4th century. Fecal transplant, known as “yellow soup”, was used as a treatment for diarrhea and food poisoning [14]. In modern science, FMT was reported as a treatment for pseudomembranous colitis for the very first time in 1958 [15]. Up till now, FMT has been widely used for *Clostridioides difficile* infections [16,17,18]. FMT has also been extended to treat other diseases, including those of the GI tract [19]. However, its therapeutic potential remains a controversial topic, especially for the treatment of extraintestinal disorders [20]. Multiple studies have suggested the role of gut microbiota and its metabolites in the onset of autoimmune disorders [21,22,23]. Recent studies have proposed that human oral and gut microbiota could play pivotal roles in modulations of immune system responses and the development of autoimmunity [24].

**Figure 1:**
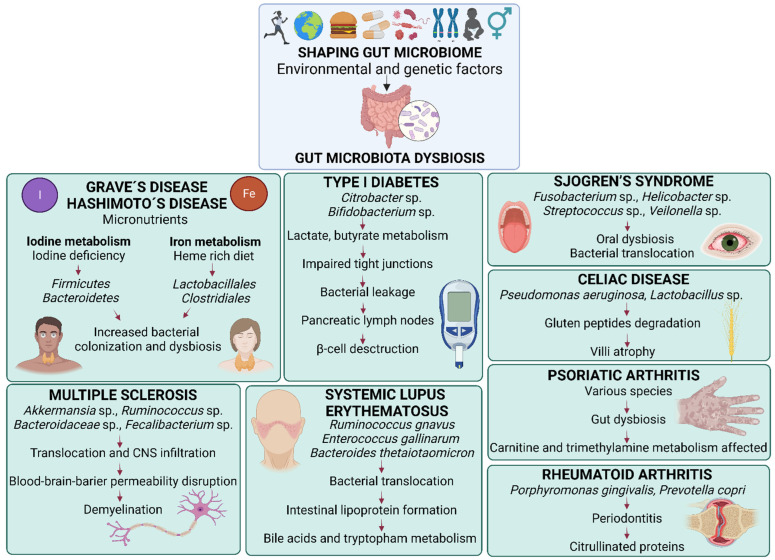
Changes in the composition of gut microbiota in selected autoimmune disease and proposed mechanisms of action. Gut microbiota is shaped by numerous environmental and genetic factors, namely age, sex, lifestyle, geographic location, diet, probiotics, drugs, genetic predisposition and previous infections. Its shift towards microbial dysbiosis increases gut barrier permeability, known as “leaky gut”, is characterized by the presence microbe-associated molecular patterns (MAMPs) and pathogen-associated molecular patterns (PAMPs), increased concentrations of lipopolysaccharides (LPS) and the release of proinflammatory cytokines in the circulation. Gut dysbiosis and increased gut permeability can play roles in the onset and progression of autoimmune diseases, namely: Graves´ disease and Hashimoto’s disease by modifying micronutrients metabolism, iodine and iron, that decrease intestinal pH; type I diabetes by bacterial translocation into pancreatic lymph nodes affecting β-cell destruction; psoriatic arthritis by carnitine and trimethylamine production in the gut; Sjogren´s disease by primary oral dysbiosis and following bacterial translocation via the bloodstream; Celiac disease by gluten peptides degradation and subsequent villi atrophy; systemic lupus erythematosus by lipoprotein formation, bile acid and tryptophan metabolisms; rheumatoid arthritis associated with oral gut microbiota dysbiosis, characterized by increased citrullinated proteins and anti-citrullinated protein antibodies; and lastly, multiple sclerosis by bacterial translocation and infiltration into the central nervous system affecting the blood-brain barrier and demyelination processes. In many of the mentioned disease conditions, the amount of Th1, Th2, Th17 and CD4^+^ T cells is increased, and the amount of short chain fatty acids and Treg cells is decreased.

This review aims to summarize the current knowledge about gut microbiota dysbiosis and the potential of FMT in studying the pathogeneses and therapies of autoimmune diseases. Herein we discuss the extraintestinal autoimmune pathologies with at least one published or ongoing FMT study either in human or mouse models. These data are provided in extensive detailed tables summarizing the current clinical studies (Table 1), ongoing clinical trials (Table 2), therapeutic experimental studies (Table 3) and pathologic state-inducing studies (Table 4) in which FMT has been applied as a treatment or inductor of autoimmune diseases. Specific changes in the composition of gut microbiota found in the selected autoimmune disorders and the proposed mechanisms leading to these changes are depicted in Figure 1.

## 2. Rheumatoid Arthritis

RA is classified as a chronic autoimmune disorder. It is characterized by progressive synovial tissue inflammation clinically manifested by pain, swelling and stiffness of joints, but also bones and cartilages [63]. RA is presumably influenced by genetic, environmental and socioeconomic factors. Although these may greatly contribute to the pathogenesis of RA, its etiopathogenesis currently has not been completely elucidated [63].

Microbial diversity changes have been reported in numerous clinical studies devoted to RA [64,65,66]. Despite all authors highlighting gut microbiota differences between RA patients and healthy individuals at different taxonomic levels, diversity and species richness information in clinical studies is inconsistent and varies. Adult American and Chinese patients’ studies reported an α-diversity decrease correlated with rheumatoid factor levels and disease duration [67,68]. The α-diversity was also significantly decreased in ACPA-positive patients compared with healthy individuals, and there were reported α-diversity differences in autoantibodies-positive and autoantibodies-negative RA patients [68]. Decreased β-diversity and species richness were reported by Sun and colleagues [1]. Surprisingly, a methotrexate treatment (MTX), one of the disease-modifying antirheumatic drugs (DMARDs), was shown to increase the α-diversity and species richness. This reestablishment could be a result of an improved health status and the restoration of gut microbial homeostasis [67]. However, no β-diversity divergence was detected in RA patients with and without MTX treatment [68]. In contrast with the above-mentioned studies, Mei and colleagues observed no α- or β-diversity or species richness discrepancies between healthy and RA individuals [69]. This was also supported by other studies [70,71]. Evidence indicates that *Porphyromonas gingivalis* could be involved in RA etiology by inducing the production of ACPAs and inflammatory processes. Moreover, *Porphyromonas gingivalis* transmission from the oral cavity into the lumen of the intestine could be a trigger of altered gut microbial composition, leading to bacterial dysbiosis, increased intestinal barrier permeability and the subsequent translocation of viable bacteria or products of their metabolism into the bloodstream [72]. Thus, FMT might be a valuable tool to restore the perturbation.

Microbiome perturbations have been described in both patients and animal models. An increase in the relative abundance of Bacteroidetes in RA patients was found, accompanied by a decreased phyla Actinobacteria, Firmicutes and Proteobacteria according to the study of 126 healthy and RA-diagnosed individuals [1]. On the contrary, an increase in Actinobacteria was reported in another study [67]. At the genus level, Sun and colleagues reported that RA patients had a significantly decreased abundance of many bacterial genera, namely *Lactobacillus*, *Alloprevotella*, *Enterobacter*, *Clostridium*, *Odoribacter*, *Enterococcus*, *Klebsiella*, *Desulfovibrio*, *Citrobacter*, *Akkermansia*, *Helicobacter*, *Rikenella*, *Staphylococcus*, *Coprococcus* and *Rhodococcus* compared with healthy individuals [1]. A total of 10 genera were less prevalent in RA patients, including *Enterococcus*, in a randomized study of the Chinese population receiving either traditional Chinese medicine or oral leflunomide treatment. In contrast, this study reported 16 genera to be more prevalent in RA patients, for instance, *Enterobacter* and *Haemophilus*, but also *Campylobacter*, *Neisseria* and *Veillonella* [69]. An abundance of the genera *Bacteroides*, *Escherichia-Shigella*, *Parasutterella*, *Flavonifractor*, *Eubacterium*, *Tyzzerella*, *Sellimonas* and *Oscillospira* was also significantly increased in RA patients [1]. However, a decrease in *Parasutterela* counts was shown in another study [69]. On the contrary, Chen and colleagues reported a decreased abundance of the genus *Faecalibacterium* in adult RA patients. This study described an increased abundance of the genera *Eggerthella* and *Actinomyces* in RA patients compared with controls [67].

Gut microbiota composition has been altered in animal models of RA, specifically in a murine model of collagen-induced colitis (CIA). An increase in Actinobacteria was reported by [56]. The ileal microbiota showed a reduced abundance of the families *Enterococcaceae*, *Lactobacillaceae* and *Streptococcaceae* and the genera *Lactobacillus* and *Lactococcus* [73].

It is vital to take into consideration the fact that a large number of RA patients are treated with at least one DMARD at the time of the study. A recently published cross-sectional clinical study declared that intestinal bacterial counts and bacteria-related markers were affected in patients treated by MTX 7.8 ± 0.3 mg/week [72]. Correspondingly, a study screening pharmaceuticals against representative bacterial isolates of the gut, including DMARDs MTX and leflunomide, revealed that MTX affected the growth of 12 bacterial species from eight genera; specifically, *Bacteroides*, *Clostridium*, *Eubacterium*, *Lactobacillus*, *Roseburia*, *Ruminococcus*, *Streptococcus*, *Veillonella* and leflunomide inhibited the growth of two genera, *Dorea* and *Ruminococcus* [74]. Lastly, gut microbiota changes were reported over time in RA patients with leflunomide and traditional Chinese medicine treatment [69]. Knowledge of microbial composition changes in RA patients compared with what is considered healthy gut microbiota could serve as a potential target for treatment and the determination of susceptibility via the detection of bacteria-specific relative abundance discrepancies. Yet, there is much to be elucidated, since the precise role of each individual species in human physiology and pathology is unknown.

The successful FMT procedure was reported in the case report of a 20-year-old woman as well. Fecal suspension from an 8-year-old healthy donor was administered via colonoscopy, and no adverse effects were observed during or after the FMT procedure [11]. Despite its safety, its potential in anti-rheumatic treatment is controversial, and the impact of FMT on RA progression varies across studies. Physical changes present in experimental murine arthritis models, especially cartilage alterations, paw deformities, increased concentrations of tissue inflammatory mediators and the activation of CD4/CD8^+^ T-lymphocytes were described in germ-free mice that were exposed to FMT originating from TNFΔARE^+/−^ donors exhibiting spontaneous RA. These changes were described together with behavioral modifications and occult bleeding, with gut tissue disruption pointing out the complex interconnections between the gut microbiota, immune system and gut-brain axis [55]. Similarly, depression-like behavior was reported in a collagen-induced arthritis murine model after the antibiotic treatment followed by FMT from RA patients. In addition, the administration of FMT resulted in an increased percentage of CD3e^+^ and CD4^+^ T-lymphocytes in Peyer’s plaques and the spleen, an increased index of the Th1/Th2 cells and decreased CD25^+^ and FOX3^+^ Treg cells in the antibiotics-treated DBA/1J murine recipients. Correspondingly, some bacterial taxa, for instance *Bacteroides* and *Phascolarctobacterium*, showed a negative correlation with the Th1/Th2 index and positive correlations with a decreased percentage of Treg cells in both Peyer’s plaques and the spleen [56]. In an animal model of adjuvant-induced arthritis, gut microbiota was modulated indirectly via tuna elastin peptides. The daily intake of tuna elastin peptides resulted in the significant downregulation of inflammatory cytokines and the upregulation of the immunosuppressive cytokines in the serums, bones and colons of the experimental mice. This positive effect of tuna elastin peptides was transmitted by FMT administration from treated recipient mice into donor ones [46]. Comparably, the inflammation was not transmitted from TNFΔARE^+/−^ donors to recipient mice colonized with bacteria compared with germ-free murine recipients, suggesting healthy mice microbiota preserve their healthy balance and possess resistance against dysbiosis arising from unbalanced FMT donors [55]. Additionally, Pu and colleagues proposed twelve promising RA biomarkers, from which seven were microbial taxa, namely *Actinobacteriota*, *Coriobacteriales*, *Coriobacteria*, *Enterorhabdus*, *Eggerthellaceae*, *Lachnospiraceae bacterium 28-4*, and *Eubacterium xylanophilum group*, highly prevalent in the group of mice that received FMT from RA patients compared with mice with FMT from healthy individuals [56]. Presumably, the first case report applying FMT as a treatment for the refractory RA patient was published by Zeng and colleagues. The FMT treatment led to a decrease in rheumatoid factor, disease activity score-28 and an improvement of the health assessment questionnaire [11]. Oral inoculation of *Porhyromonas gingivalis* modified the gut microbiome in an experimental model of arthritis. Severe joint swelling was shown in laminarin-induced and *Porhyromonas gingivalis*-inoculated mice in combination with laminarin-induced experimental arthritis after FMT administration. The maximum observed arthritis score was significantly higher in mice that received FMT compared with non-FMT mice [57]. FMT was applied to prevent *Clostridioides difficile* relapse after vancomycin treatment. Surprisingly, *Clostridioides difficile* (strain VPI 10463) infection attenuated experimental CIA arthritis without any antibiotic treatment and FMT administration more effectively. Contrarily, FMT treatment led to arthritis scores comparable to rodents in which only CIA was induced. The results illustrated that gut microbiota is involved in arthritis acceleration, but surprisingly suggested that an aggressive VPI 10463 strain attenuated its progression [58].

FMT as a potential novel treatment is now being applied in numerous extraintestinal disorders. Other than RA, recent reviews analyzed the connection between the gut microbiota and other musculoskeletal diseases and proposed that FMT could represent a potential intervention in juvenile idiopathic arthritis and osteoarthritis [75,76].

## 3. Psoriatic Arthritis

Psoriatic arthritis (PsA) refers to the chronic and progressive immune-mediated inflammatory disorder characterized by heterogeneous clinical features, e.g., joint pain, stiffness and swelling that oftentimes occur in adult people with a several-year psoriasis history [77,78]. The risk factors include an interplay of genetic susceptibility, immune responses and environmental factors, including our own microbiota. Knowledge of these factors would help to understand the pathogenesis of PsA and to streamline the treatment [79].

To our knowledge, only two studies investigated gut microbiota composition directly in PsA patients. However, numerous studies focus on microbiota in the broader context of other inflammatory arthropathies [77,80,81]. Distinctive microbial features were observed in the clinical study of 16 PsA patients, 15 skin psoriasis patients and 17 healthy respondents. As proposed in the results, the overall gut microbiota composition of PsA patients was significantly different from healthy individuals, as well as from patients with psoriasis. The relative abundance of *Clostridium*, *Akkermansia* and *Ruminococcus* were decreased in PsA compared with other groups [2]. The study of nine PsA patients and their matching no-PsA controls was recently published. PsA patients displayed a shift in the microbial diversity and an increased number of observed bacterial species compared with the no-PsA control group. At the phylum level, the most prevalent PsA was Bacteroidetes, followed by Firmicutes, Proteobacteria and Actinobacteria. Moreover, gut microbiota of the PsA was similar to the no-PsA group, except for the phylum Actinobacteria that was significantly more abundant in the PsA group. Numerous significant differences across the groups were proposed at various taxonomical levels [78].

Up to the present, FMT has been applied in clinical studies. When assessing the safety, a double-blind parallel study of active peripheral psoriatic arthritis patients undergoing FMT via one duodenal treatment showed it to be acceptable and safe. Mild adverse effects were present, mainly including abdominal discomfort, nausea and vomiting. However, no life-threatening effects were recorded [12]. According to another study published from the same research group, the FMT treatment efficiency was higher in the placebo group compared with the FMT group, 81% vs. 40%, respectively. A health assessment questionnaire disability index improved more in the placebo group than in the FMT group. These results suggest that FMT may not be exclusively effective in the treatment of psoriatic arthritis, but further studies are needed [27]. To our knowledge, a randomized, placebo-controlled FMT trial has been recently completed in patients with active psoriatic arthritis non-responding to MTX treatment [38]. FMT appeared not to cause life-threatening adverse effects, but simultaneously was not successful for frequently compared with the placebo group [27].

Taken together, the scarcity of studies regarding FMT therapy in PsA patients does not allow for the drawing of any firm conclusions. Despite that, additional research in this area has reasons to be optimistic, as the possibility of FMT intervention in PsA cannot be ruled out.

## 4. Multiple Sclerosis

The term “multiple sclerosis” (MS) describes a multifocal inflammatory disease of the central nervous system characterized by immune-mediated demyelination and progressive neurodegeneration arising from autoimmune reactions to an individual´s autoantigens. Most patients suffer from the relapsing-remitting type of MS (RRMS) manifested by increasing neurologic symptoms followed by periods of recovery [82,83]. The onset of MS is not currently fully understood. It is believed that both genetic and environmental factors are probably involved in the development of MS [84].

Various studies reported changes in the gut microbiota composition of MS patients compared with healthy individuals [3,83,85,86]. However, similarly to RA, divergent results are being reported. No differences in the α-diversity, β-diversity and principal component analysis group-specific clustering were reported in the longitudinal multi-omics study of 49 MS patients and their matched healthy control individuals [3]. Similar results were described in the study of 30 Italian MS patients and their household relatives by the 16S rRNA gene sequencing analysis [83]. Principal component analysis showed no differences in the study of 15 patients with an RRMS diagnosis as well [85]. Comparably, another study of the RRMS and progressive MS patients described no differences in evenness, but increased phylogenetic diversity, Shannon diversity and richness in all participating patients compared with healthy controls. Additionally, this study proposed β-diversity differences between healthy participants and patients, but not between RRMS and progressive MS patients [87]. Furthermore, α-diversity and β-diversity were shown not to be in correlation with MS activity in the study of 60 children diagnosed with pediatric-onset MS [88]. When analyzing the most prevalent bacterial phyla of the gut microbiome, sequencing analysis showed that Firmicutes were less abundant and Bacteroidetes with Proteobacteria more abundant in MS patients compared with their control household relatives. No significant difference was reported in the phylum Actinobacteria between the study groups [83]. At the family level, five families were significantly more abundant, for instance, *Christensenellaceae*, *Desulfovibrionaceae* and *Clostridiales*, in the study of Italian patients. Comparably, four families were more prevalent in the control group, specifically *Bacteroidaceae*, *Tannerellaceae*, *Veillonellaceae* and *Burkholderiaceae* [83]. The gut microbiota of RRMS patients differed from healthy controls in the relative abundance of the families *Lachnospiraceae* or *Ruminococcaceae* in numerous studies [3,83,85,86]. The study from Cantoni and colleagues proposed a significant decrease in the relative abundance of genera *Faecalibacterium*, *Bifidobacterium*, *Clostridium*, *Bacteroides*, *Parabacteroides*, *Escherichia*, *Prevotella*, *Anaerostipes* in RRMS patients according to multiple comparison correction by false discovery rate and whole genome sequencing. From the above-mentioned taxa, six species with significantly decreased abundances showed immune-modulatory properties [3]. Discrepancies in the relative abundances were reported also in genera *Ezakiella*, *Hungatella*, *Roseburia*, *Shuttleworthia*, *Porphyromonas*, and *Bilophila* in the study of 15 RRMS patients [85]. Contrarily, the species *Bacteroides fragilis* was significantly more present in the MS patients according to a study of 30 Egyptians by quantitative SYBR Green real-time PCR assay [89]. The species *Clostridium bolteae*, *Ruthenibacterium lactatiformans* and genus *Akkermansia* were found increased in abundance in both RRMS and progressive MS. Some *Clostridium* species possess a correlation with the higher expanded disability status scale and fatigue scores [87]. Likewise, Horton and colleagues described five taxa associated with disease activity outcomes, specifically clinical relapses, gadolinium-enhancing lesions and T2-hyperintense lesions [88]. Moreover, Cox and colleagues described that disease status, BMI, race and sex had the highest effects on gut microbiota composition, respectively [87]. Not only the gut microbiota, but also microbiota-related metabolic potential, lipopolysaccharide and starch metabolites were altered in MS patients, according to a study of 37 respondents from the Canadian Pediatric Demyelinating Disease Network’s study [90]. These findings suggest that the human gut composition of MS patients has its own specific features that may be involved in MS progress [3,83].

To investigate whether the gut microbiota modifications would influence the induction and progress of an acute demyelinating process in the central nervous system, mice were orally treated with an antibiotic cocktail. The authors proposed that an antibiotics-induced reduction of gut microbiota modulated peripheral immune tolerance and protected against experimental autoimmune encephalomyelitis (EAE) in SJL/J and C57BL/6 mice [91].

In an experimental murine study, the authors investigated whether gut microbiota modifications could impact the intermittent fasting and its protective effect on the severity of EAE. Female germ-free C57BL/6J mice were treated with FMT, and EAE was induced. The results showed a significant reduction of cord pathology and ameliorated disease severity, indicating that gut microbiota could be related to intermittent fasting and thus indirectly have immunomodulatory effects [44]. Another study indicated shifts of gut microbiota towards healthy controls; however, these were not statistically significant. Despite that, a significant decrease of 13 taxa was illustrated in the EAE group post-FMT treatment. On the contrary, 17 taxa were significantly increased after the treatment. Numerous bacteria-specific negative and positive correlations were described with EAE scores and EAE cumulative scores, suggesting that specific microorganisms may play various roles in the pathogenesis of EAE. Moreover, the FMT treatment resulted in the delayed onset and amelioration of EAE, e.g., reduced clinical scores, cumulative disease scores, increased expression of a tight junction protein, Claudin 5 (crucial for blood-brain barrier and myelin basic protein) and a decreased expression NF-L neurofilament light chain protein responsible for axonal damage when released as compared with saline-treated controls [45].

The therapeutic potential of FMT was analyzed in a clinical study of nine RRMS patients. Of these, four patients received early intervention monthly for 6 months and were monitored for 6 months after FMT treatment, whereas five respondents received late intervention and were tested for six months prior to intervention. The results showed no statistically significant changes of pro-inflammatory (IL-6, IL-15, GM-CSF), regulatory (IL-10), Th17 cells (IL-17A, IL-17F, IL-21, IL-22) and Th1 cells (INFγ, IL-2, TNFβ) cytokines in plasma were observed before and after the treatment across both groups. Abnormal small intestinal permeability was reported in two out of five patients at the baseline. However, this improved after the FMT treatment. Donor-specific alterations of gut microbiota were seen. The α-diversity and β-diversity between the donor and MS patients were not significantly modified as a consequence of high intraindividual variability. Other than one patient, no FMT-related adverse effects were recorded. Frequent adverse events included nausea, vomiting or abdominal discomfort [13]. The clinical case of a 48-year-old Caucasian male with active RRMS showed an increase in short-chain-fatty-acids (SCFA) genomic pathways across the time of the study, suggesting that FMT could be related to the SCFA metabolic pathways in RRMS [26]. The relative abundance of microbial SCFA pathway gene content displayed a positive correlation with serum brain-derived neurotrophic factor, a neurotransmitter modulator that influences the survival and plasticity of neurons. One specific butyrate-producing bacteria, *Faecalibacterium prausnitzii*, was significantly elevated post-FMT. Moreover, butyrate propionate, total SCFA and total-butyrate-to-total SCFA ratio concentrations were significantly increased in two out of five post-FMT measurements [26].

In conclusion, a number of publications described the abundance and composition of bacterial taxa in MS. Although significant in its own aspect, the contribution of these results to the causality is limited. Rather, these observations provide a ground for future studies aimed at exploring the precise role of gut microbiota in MS development.

## 5. Type 1 Diabetes

According to a study from 2019, the worldwide prevalence of type 1 diabetes mellitus (T1D) is more than a million people. The highest prevalence was found in Europe and North America, although regional and economic differences between the continents must be considered.

Numerous studies showed differences in composition of gut microbiome of T1D patients compared with healthy controls. In particular, an increase in the abundance of Bacteroidetes in T1D patients was found, accompanied by a decrease in Firmicutes [4,92,93]. Interestingly, the opposite result was reported by Pellegrini and colleagues [94]. These results were corroborated by a recent Chinese study, in which the abundance of multiple *Bacteroides* genera negatively correlated with fasting blood glucose, while other genera such as *Blautia*, *Eubacterium*, *Anaerostipes* or *Dorea* positively correlated with fasting blood glucose. The authors even proposed that some of the genera could be used as a non-invasive biomarker to distinguish T1D patients from healthy controls [95]. Qi et al. documented an overall lower bacterial richness in T1D patients with increased genus *Blautia* and, conversely, decreased genera, including *Haemophilus*, *Lachnospira* or *Dialister* [96]. Other than gut colonization with more harmful bacteria, dysbiosis may have other consequences, including, for instance, a decrease in SCFA concentration [14] or even an increase in intestinal permeability [97]. Dysbiosis may thus modulate the gut microbial and structural profile into a pre-inflammatory state which may share similarities with gastrointestinal disorders. Moreover, it may be worthwhile to investigate first-degree relatives of T1D patients, as a higher and lower proportion of *Bacteroides* and *Prevotella*, respectively, was found in first-degree relatives positive for multiple autoantibodies [98].

Results of FMT studies in mice are controversial. While non-obese diabetic mice cross-fostered by non-obese diabetes resistant mothers showed improved mucus production [99], another study found neither a delay in diabetes onset nor reduction in intestinal inflammation or the amount of regulatory T cells in non-obese diabetic mice cohoused with C57BL/6 mice [100]. Interestingly, even the exact composition of metabolites including short chain fatty acids (SCFA) may be important, since protection against diabetes was achieved after the transplantation of microbiota shaped by a high acetate as opposed to butyrate diet [101]. Finally, a study by de Groot and colleagues revealed that FMT from healthy donors halted, or at least significantly slowed, T1D progression in recent onset (<6 weeks) T1D patients [29]. Thus, it is clear that fecal microbiota is involved in the onset and progression of T1D, although possibly rather as a contributor than the main player. This could be hypothesized based on the results of a study which transplanted fecal microbiota from T1D patients to germ-free non-obese diabetic mice. The adoption of the T1D gut microbiota was successful; however, the rate of pancreatic beta cell loss was not replicated [62], suggesting the presence of some other factors which could influence metabolic status. Additional studies are warranted to elucidate the complex mechanisms and interactions which occur in the gut and affect other organs of the body.

Apart from the controversial nature of results regarding the involvement of gut microbiota in the pathogenesis of T1D, it seems that SCFA composition and concentration may be equally important. Additional studies recruiting first-degree relatives of T1D patients may serve to clarify the role of certain bacterial taxa in the development and progression of T1D.

## 6. Systemic Lupus Erythematosus

Systemic lupus erythematosus (SLE) can be classified as an inflammatory autoimmune disease affecting multiple organs. Most commonly, it manifests as skin rashes found in various areas around the body. Despite the proposed genetic and environmental triggers, the exact cause is still unknown.

There are a number of studies which show distinct changes in microbial composition of the gut microbiota in mice, as well as in humans. For instance, microbial dysbiosis has been documented in MRL/lpr mice, a commonly used lupus-prone strain. These mice had a marked reduction in the family *Lactobacillaceae* and, conversely, an overabundance of the *Lachnospiraceae* family [102]. Moreover, diet supplementation with retinoic acid increased the abundance of *Lactobacillaceae*, which in turn translated into an amelioration of the symptoms. A similar positive effect on the same murine strain was achieved using antibiotics (a mixture of ampicillin, neomycin, metronidazole and vancomycin), although it should be noted that the authors used only female mice [103]. Interestingly, even various brands of commercially available murine diet may have a profound impact on the composition of intestinal microbiota (*Lachnospiraceae* in particular) in MRL/lpr mice [104]. Indeed, it may not be feasible to completely remove this diet-mediated effect on the gut microbiota; however, it should be kept in mind that diet may either contribute to or, conversely, partially negate the observed results.

Wang et al. found a lower ratio of Firmicutes/Bacteroidetes in 6-week-old MRL/lpr mice caused by a drop in *Peptostreptococcaceae* belonging to Firmicutes phylum and, in contrast, a rise in *Rikenellaceae*, a member of Bacteroidetes phylum. This microbial shift was accompanied by an increase in colonic oxidative stress, lower tight junction protein zonula occludens 2 and increased inflammatory markers such as nuclear factor kappa B (NF-κB) and interleukin (IL)-6 [105]. Indeed, a decreased Firmicutes/Bacteroidetes ratio was reported in Spanish patients [106] and Chinese SLE patients, along with the enrichment of certain genera such as *Klebsiella*, *Prevotella* or *Flavonifractor* [107]. Although a study by Luo et al. did not confirm the difference in the Firmicutes/Bacteroidetes ratio between SLE patients and controls [5], a more recent publication which studied Egyptian SLE patients found lower Firmicutes/Bacteroidetes, with a concomitant decrease in genus *Lactobacillus* as well [108]. Using NZB/W F1, another murine strain displaying SLE-like symptoms, presensitized with human cytomegalovirus pp65 peptide, yielded higher abundance of the families *Saccharimonadaceae*, *Marinifiaceae* and *Desulfovibrionaceae* and genera including *Roseburia*, *Odoribacter* and *Desulfovibrio* [61].

Experimental evidence suggests that the SLE-like microbial profile may be transported via FMT. Germ-free C57BL/6 mice given FMT from SLE-prone mice led to changes in immune cell distribution in recipients and, more importantly, upregulated expression of lupus susceptibility genes [60]. FMT may also be beneficial to mediate the effect of corticosteroids such as prednisone. In a study by Wang and colleagues, gut microbiota from prednisone-treated MRL/lpr mice decreased the abundance of genera *Ruminococcus* and *Alistipes*. Interestingly, the authors reported that prednisone-treated gut microbiota did not exhibit side effects when transplanted to blank MRL/lpr mice [49]. One week of FMT was able to alleviate gut dysbiosis caused by prior antibiotics administration and suppress SLE progression [50]. It was found that SLE-like symptoms bestowed upon the host by donor gut microbiota could be due to the promotion of lymphocyte activation and Th17 differentiation from naïve CD4^+^ lymphocytes [109]. It is noteworthy that even human-to-mouse inter-species FMT is capable of eliciting SLE-like symptoms and gene expression in the recipient species [61], pointing towards the similarities in gut microbiota composition between the two species.

Taken together, distinct changes in the microbiota have been observed in both SLE-prone mice and SLE patients. Remarkably, the microbial profile can be transported between the two species, opening new possibilities of various study designs to be explored.

## 7. Celiac Disease

Celiac disease (CeD) is defined as an autoimmune disease which arises as a response to the ingestion of gluten, a component of wheat, barley and rye. Although the exact cause behind the intolerance of gluten is unknown, environmental triggers contributing to an already pre-existing genetic susceptibility in some individuals is a generally accepted hypothesis.

A number of studies uncovered differences in fecal microbiota composition between CeD patients and healthy controls. For example, Collado and colleagues reported a higher abundance of genera *Bacteroides*, *Clostridium* and *Staphylococcus* in pediatric CeD patients compared with healthy age-matched controls. Fluorescence in situ hybridization revealed higher counts of bacteria such as *Clostridium histolyticum* or *Eubacterium rectale* implicated in intestinal infection and even in the progression towards colorectal cancer [110]. In contrast, there was a trend toward a higher amount of the genus *Bifidobacterium* in healthy controls [6]. This result was later confirmed in CeD adults [111]. Apart from a reduction in *Bifidobacterium* in CeD patients with poly-autoimmunity, Bibbò et al. found a lower abundance of *Bacteroides*, *Ruminococcus* and *Veillonella* genera. Additionally, a negative and positive correlation of CeD duration with the abundance of Firmicutes and *Odoribacter*, respectively, was detected [112]. Based on their results, Olivares and colleagues hypothesized a role of gut microbiota in CeD predisposition in infants later in life [113]; however, another study comparing children who later developed CeD with healthy controls did not find supportive results to corroborate the hypothesis [114]. Another study documented abundant *Proteobacteria* and *Neisseria* in active CeD patients, with treated patients showing a transition in microbial composition between active disease and a healthy state. Interestingly, the salivary microbiota was reported to match the mucosal microbiota more closely compared with the stool, showing the potential for salivary diagnostics [115]. An in-depth review on salivary microbiota in CeD patients was published by De Angelis et al. [116].

Given the genetic background in CeD, it stands to reason to look at the fecal microbiota of not only CeD patients but their first-degree relatives as well. Although 16S rRNA sequencing did not reveal significant differences in microbial diversity between CeD patients, their relatives and controls, certain amplicon sequence variants were affected. For instance, genera including *Parvimonas*, *Granulicatella* and *Bifidobacterium* were more abundant in the duodenal microbiota of CeD relatives. In contrast, the duodenal microbiota of CeD patients was enriched in *Megasphaera* and *Helicobacter* compared with the microbiota of their relatives. It is noteworthy that the authors found higher differences in duodenal compared with fecal samples. In addition, there was a lower amount of *Akkermansia* and *Dorea* in both CeD patients and their relatives compared with the controls. These alterations of the microbiota could lead to a decreased ability of CeD patients microbiota to metabolize gluten compared with their relatives [117].

So far, intervention studies have been rather scarce. Four weeks of a gluten-free diet yielded a higher abundance of the genus *Actinobacillus* and the family *Ruminococcaceae* in the duodenal and fecal microbiota of non-celiac gluten sensitivity (NCGS) patients compared with CeD patients, with a concomitant higher amount of *Novispirillum* in CeD patients [118]. Oat ingestion in CeD and NCGS patients did not lead to microbial dysbiosis, and the concentrations of SCFA such as propionate or butyrate were comparable among the groups [119]. Interestingly, it could seem that fecal microbiota may not be directly linked with gluten ingestion, as a two-week gluten challenge did not cause significant microbial changes in CeD and NCGS patients compared with the pre-existing state [120]. Before the effect of gut microbiota on gluten sensitivity can be dismissed, however, recent advances in cell pathways must be considered. When non-obese diabetic mice expressing DQ8, a CeD susceptibility gene, were given a high-tryptophan gavage as well as a *Lactobacillus reuteri* gavage, the production of aryl hydrocarbon receptor (AhR) ligand was increased and the AhR pathway was activated. Patients with active CeD showed a lower production of AhR ligand by the fecal microbiota compared with healthy controls [121].

To the best of our knowledge, the study of FMT effects in CeD has been limited to a case report in which a patient with refractory celiac disease type II and recurrent *Clostridioides difficile* infection received an FMT. The procedure not only cured the *Clostridioides difficile* infection, it also mitigated the CeD symptoms. At the follow-up after six months, no villous atrophy in duodenal biopsies was present [25]. While this report looks promising, a larger study involving multiple cohorts of patients is needed to confirm these results. Notably, there is an ongoing clinical trial by the Chinese University of Hong Kong aimed at exploring the safety and efficiency of FMT in various gastrointestinal and metabolic disorders, including *Clostridioides difficile* infection or CeD (Safety and Efficacy of Fecal Microbiota Transplantation, NCT04014413).

Although studies focused on the composition of fecal microbiota bring vital information regarding gut microbial dynamics, they offer little to elucidate the causality between microbiota and the development of CeD. Apart from that, results from such studies are often challenging to compare and interpret [122]. This is partly due to the lack of sufficient animal models; however, recent developments look promising [123]. In addition, research aimed at the differences in cellular pathways, particularly the AhR pathway, may help to uncover new crucial insights into CeD pathology.

## 8. Hashimoto’s Disease

Hashimoto´s disease (HD), or Hashimoto´s thyroiditis, represents an organ-specific autoimmune disease, also known as chronic lymphocytic thyroiditis. In HD pathogenesis, intrathyroidal mononuclear cells are infiltrated and autoantibodies against thyroglobulin and thyroid peroxidase are produced, inducing organ enlargement, gland fibrosis, decreased thyroid hormone and, finally, reduced metabolic activity in more tissues [124,125]. It is considered one of the most common autoimmune diseases worldwide, reaching a prevalence of 10–12% in adults between 30–50 years of age, more often affecting females [126]. The etiology of HD remains unclear at the moment. Similarly, as in the diseases debated above, the interplay of genetic predisposition and epigenetic factors could have an impact on the onset and progression of HD [127]. A detailed interconnection of HD, immunity and gut microbiota is described in the review article by Virili and colleagues [128].

Some studies have proposed compositional modifications and bacterial dysbiosis in the gut microbiota of HD patients, suggesting that specific bacterial overgrowth and its impact on gut-thyroid axis may play a pivotal role in health. Compared with healthy controls, bacterial richness and diversity were significantly decreased in HD patients with both euthyroidism and hypothyroidism [7]. Whereas the diversity and richness are oftentimes very similar compared with the gut of a healthy population, the overall compositional structure varies. Partial least squares-discriminant analysis showed a disturbance in the microbiota composition of HD patients compared with to healthy individuals. Interestingly, there were no significant gut microbiota differences between the HD group and Graves‘s disease patients [129]. A cross-sectional study of 28 HD patients and 16 matching controls described comparable bacterial richness and diversity indices [127]. α-diversity estimators, ACE, Chao1 and observed species, were significantly increased in HD, but surprisingly, no aberrations in the Simpson and Shannon diversity index were presented between groups in the study of 29 HD patients and 12 healthy volunteers [130]. However, the study of primary hypothyroidism patients revealed a significant gut microbiota difference in α-diversity and β-diversity between patients and healthy individuals [54].

A sequencing analysis proposed group variances at several taxonomic levels [127]. Bacterial phyla Firmicutes and Actinobacteria were increased in the fecal matter of HD individuals, whereas phyla Bacteroidetes and Proteobacteria were decreased [127]. In contrast, Proteobacteria were the most prevalent in HD patients, followed by Graves’ disease patients and the control group in the study of the 27 HD patients, respectively [129]. A significantly higher relative abundance of Proteobacteria was proposed also in the study of Ishaq and colleagues, together with decreased Firmicutes and Bacteroidetes [130]. Moreover, the Firmicutes/Bacteroidetes ratio was significantly elevated in the HD patients [127]. As numerous studies show, the families *Lachnospiraceae*, *Bacteroidaceae*, *Enterobacteriaceae*, *Alcaligenaceae*, *Coriobacteriaceae*, *Erysipelotrichia* and *Bacillobacteriaceae* were highly abundant in the gut microbiota of the HD group. Contrarily, *Prevotellaceae*, *Ruminococcaceae* and *Veillonellaceae* were lowered in the HD patients group [127,129,130].

At the genus level, the relative abundance of genera *Bacteroides, Faecalibacterium, Prevotella* and *Lachnoclostridium* were decreased, whereas the *Blautia*, *Ruminococcus*, *Roseburia*, *Fusicatenibacter*, *Romboutsia*, *Dorea* and *Eubacterium* genera were significantly increased in the HD patient fecal samples [127]. A decrease in *Prevotella* has been proposed in numerous studies [130]. Four bacterial genera, *Veillonella*, *Paraprevotella*, *Neisseria* and *Rheinheimera*, were strictly present in the primary hypothyroidism patients and thus could distinguish patients from healthy individuals, as confirmed by the random forest analysis and receiver operator characteristic curve analysis. Lastly, increased serum lipopolysaccharides (LPS) concentrations were reported [54]. A systematic review and meta-analysis summarized that probiotic bacterial genera, such as *Bifidobacterium* and *Lactobacillus*, were decreased in the autoimmune thyroid diseases, whereas the species *Bacteroides fragilis* was significantly elevated compared with the controls [131].

Several taxa correlated with the host metabolism or host clinical parameters. The second cohort of a cross-sectional study of 22 patients and 11 matching controls proposed that 18 genera positively correlated with TPO-Ab or TG-Ab, while another 6 genera showed negative correlations. Moreover, the genus *Alloprevotella* showed positive correlations with free thyroxine, the genus *Fusicatenibacter* exhibited negative correlation with free thyroxine and the genus *Romboutsia* negatively correlated with serum thyrotropin [127]. Four bacterial strains were associated with a greater risk of occurrence and development of HD by affecting glutathione and arachidonic acid metabolisms. Another six bacteria were involved in the development of HD by affecting purine and pyrimidine metabolism pathways [129].

Research articles analyzing the role of FMT in the pathogenesis of HD are lacking. To our knowledge, there is a single experimental study published recently. In 20 pathogen-free BALB/c male mice, FMT delivered from primary hypothyroidism patients resulted in a progressive decrease of the serum total thyroxine concentrations compared with mice that were exposed to FMT delivered from healthy respondents, reaching a significant decrease at six weeks post-FMT. This suggests that gut microbiota alterations could influence the function of the thyroid gland in an experimental murine model. Furthermore, serum LPS concentrations, mRNA expression of occludin, junctional adhesion molecule-A and zonula occludens-3211, known as tight junctions, were decreased in the colon of FMT-HD recipients six weeks post-FMT [54]. Unfortunately, there is no ongoing clinical study focusing on the effects of FMT on Hashimoto’s disease at the moment.

To conclude, the abundance of many bacterial taxa was either positively or negatively correlated with certain clinical parameters, such as free thyroxine or serum thyrotropin. Although the relationships of bacteria with any of those parameters are far from understood, they provide a solid starting point for future research.

## 9. Graves’ Disease

Graves’ disease (GD) is defined as a thyroid gland-specific autoimmune disorder, characterized by hyperthyroidism arising from autoantibodies that activate the thyrotropin receptor. Due to this, symptoms such as weight loss, fatigue, tachycardia, heat intolerance and exophthalmos develop. The etiology is currently not clear, and the genetic and epigenetic factors are involved. The estimated annual incidence is 20 cases per 100,000 people [132,133].

Numerous clinical studies, originating mostly from China, reported microbial dysbiosis and reduced microbial diversity in GD patients [8,53,134]. Correspondingly, a decreased α-diversity and reduced Firmicutes/Bacteroidetes ratio was oftentimes described along with GD [53,132,135]. A clinical study of 36 mild and 64 severe GD Chinese patients described similarities between healthy and mild GD, but there was a significant bacterial decrease in severe GD patients [8]. Proteobacteria and *Erysipelotrichia* were increased in Chinese adult GD patient samples. Moreover, the relative abundances of *Erysipelotrichaceae*, *Lachnospiraceae*, *Alcaligenaceae* and *Christensenellaceae* was increased compared with the control and HD groups. At the genus level, *Prevotella*_9, *Ruminococcus*_2 and *Lachnospiraceae*_NK4A136_group were enriched in the fecal matter of GD patients [129]. Additionally, an upregulation of the bacteria *Bacilli*, *Lactobacillales*, *Prevotella*, *Megamonas* and *Veillonella* was shown among GD patients compared with healthy individuals in the clinical study of 37 patients [134]. Genera *Bacteroides* and *Lactobacillus* showed a higher proportion in 45 Chinese GD patients [135]. Moreover, genus *Bacteroides* showed a negative correlation with the pathogenic bacteria *Prevotella*_2 in healthy individuals, but not in the GD group. A negative correlation was reported between genera *Dialister* and *Streptococcus* in healthy individuals, but showed as a positive in GD patients [53]. At the species level, a decrease in relative abundances of *Akkermansia muciniphila*, *Bifidobacterium adolescentis*, *Butyricimonas faecalis* and *Faecalibacterium prausnitzii* was described in severe GD. In contrast, species *Eggerthella lenta*, *Fusobacterium mortiferum*, *Veillonella parvula*, *Streptococcus parasanguinis* and *Streptococcus salivarius* were more prevalent in severe GD group compared with controls [8]. Some taxa were shown as potential biomarkers of GD. Specifically, bacterial genera *Bacillus*, *Blautia*, *Bacteroides*, *Ornithinimicrobium*, *Alistipes* and *Prevotella* were proposed as potential markers to distinguish GD from the healthy people [53,129,135]. The compositional differences in gut microbiota could be related to the pathogenesis of GD. One of potential markers, *Bacteroides*, may play a key role in its development and progression.

Several studies aimed to modify gut microbiota to determine its role in thyroid autoimmunity [51,52,53]. In the experimental murine model, FMT was applied to analyze the potential of the gut microbiome in the function of thyroid hormones that influence host thermogenesis and energy metabolism. Hyperthyroid gerbils were administered FMT from control experimental animals or sterile saline. On the other hand, control gerbils were given hyperthyroid gerbils´ delivered FMT or sterile saline. The results showed that FMT led not only to gut microbiota alterations between the groups, but also that control FMT resulted in a greater decrease of T3 and T4 hormones concentrations, increased liver expression of type 2 deiodinase and a better recovery of hypothyroid-induce resting metabolic rate back to normal. These revealed that changes in the gut bacterial composition may diminish hyperthyroid-induced thermogenesis in the gut microbiota-thyroid axis [51]. Half of the GD-diagnosed patients have Graves’ orbitopathy. In experimental female BALB/c mice, four different treatments were applied to investigate the bacterial potential in disease heterogeneity: the antibiotic treatment provided by vancomycin, a probiotic treatment consisting of *Lactobacillus* and *Bifidobacterium* strains, a FMT treatment pooled from six Graves’ orbitopathy patients and the control group receiving water. Each group was immunized with the plasmid encoding human thyrotropin receptor (TSHR)-A subunit or the control plasmid (βgal). The results indicated compositional differences of microbiota across the groups. Regarding FMT, the BALB/c recipients inherited patterns of donor´s unbalanced microbiota, resulting in an increased disease severity. The FMT-TSHR group was characterized by a reduction in Shannon diversity, but a significantly increased richness indices compared with control-TSHR mice and a decreased abundance of *Bacteroides* compared with the control and FMT-βgal mice. The overall results of all four groups proved the role of microbiota in GD [52]. Human-to-mouse FMT was performed in another research article. Pathogen-free BALB/c mice were transplanted fecal matter from GD or a healthy population. Immunization was performed by adenovirus transfection of TSHR or blank vector. The FMT from either healthy or GD patients showed similar results, i.e., no effect on the thyroid gland after a blank immunization. However, FMT from GD patients in TSHR-transfected mice resulted in increased GD incidence from 28.6% to 73.3%, increased serum concentrations of total thyroxine, thyroglobulin antibodies and IL-17A and decreased serum concentrations of IL-10 [53].

These results proposed that gut microbiota may be implicated in the pathogenesis of GD, but it is likely not the only triggering element. Thus, it can be established that the co-occurrence of more pathogenic factors is needed for the development of GD.

## 10. Sjogren’s Syndrome

Sjögren’s syndrome (SS) is classified as an inflammatory autoimmune disorder with lymphocytic infiltration, either as primary SS or secondary SS in association with RA, SLE or another autoimmune rheumatic disease. Affecting the lacrimal and salivary glands, but also other exocrine glands, common symptoms could be dry eyes or keratoconjunctivitis sicca, salivary flow, and positive serum anti-Ro antibodies or rheumatoid factor. Primary SS belongs to the common systemic autoimmune diseases, reaching a prevalence of 0.1 to 0.6%, affecting predominantly women [136,137]. Intricate relationships between genetic and epigenetic factors can impact the onset of primary SS.

Gut microbiota displayed α-diversity and β-diversity differences between SS patients with immune-mediated dry eye syndrome and healthy individuals [28]. The Shannon index, ACE index, Chao index and Sobs index were reduced and the Simpson index was elevated in the primary SS group before treatment in the study of 16 Chinese patients [9]. Schaefer and colleagues reported an overall diversity decrease [59]. Firmicutes were described to be the most abundant phyla, followed by Bacteroidetes, Proteobacteria and Actinobacteria [138,139]. Compared with healthy individuals, Bacteroidetes and Proteobacteria were elevated, whereas Firmicutes and Actinobacteria decreased in SS patients compared with healthy controls [139]. Comparably, no differences in the Bacteroidetes/Firmicutes ratio were recorded according to a clinical study published by Mendez and colleagues [138]. A sequence analysis at the family level proposed that the relative abundances of *Actinomycetaceae*, *Eggerthellaceae*, *Lactobacillaceae*, *Akkermansiaceae*, *Coriobacteriaceae* and *Eubacteriaceae* were statistically elevated compared with healthy individuals [138]. Similarly, Wu and colleagues reported an increase in *Bacteroidaceae*, *Ruminococcaceae*, *Veillonellaceae* and *Enterobacteriaceae* in SS patients [9,139]. Another study reported an increase in *Clostridiaceae*, *Prevotellaceae*, *Rikenellaceae*, *Odoribacteraceae* and *Veillonellaceae* as well, but a decrease in *Porphyromonadaceae* and *Bifidobacteriaceae*. A completely different composition of gut microbiota was described in some other studies. Specifically, a decreased relative abundance of *Ruminococcaceae*, *Lachnospiraceae* and *Bacteroidaceae* was found in SS patients compared with healthy controls [138,139]. In a study of 16 Chinese women, the bacterial genera *Bifidobacterium*, *Bacteroides*, *Escherichia-Shigella*, *Faecalibacterium* and *Prevotella* were significantly more prevalent in the SS group. Moreover, the genus *Faecalibacterium* was not detected in healthy subjects at all. However, the composition changed after therapy, indicating a shift to eubiosis [9]. An increase in *Prevotella* was shown in another article by Cano-Ortiz and colleagues, together with genera *Megasphaera*, *Escherichia*, *Clostridium*, *Enterobacter* and *Streptococcus*. Conversely, the genera *Bacteroides*, *Bifidobacterium* and *Faecalibacterium* were decreased in other clinical studies, accompanied by *Alistipes*, *Dorea*, *Blautia*, *Lachnospira*, *Roseburia* and *Ruminococcus* [138,139]. The relative abundance of *Veillonella* and *Parabacteroides* is controversial. The study by Cano-Ortiz and colleagues reported an increase in the relative abundance of *Veillonella* and a decrease of *Parabacteroides*, whereas Mendez and colleagues described a decrease of *Veillonella* along with the increase of *Parabacteroides* [138,139]. Furthermore, specific bacterial strains could help to distinguish SS patients from healthy subjects, namely *Bacteroides caecimuris*, *Mediterranea massilliensis*, *Bacteroides coprophilus*, *Clostridium*_sp_7_3_54FAA and *Bifidobacterium bifidum*. All of these, except for *Bifidobacterium bifidum*, were significantly decreased in SS patients compared with the healthy population. The abundance of all of the mentioned species correlated with disease severity [59]. Putting this in the context of our findings, the variability of gut microbiota is extensive, and changes in SS are oftentimes inconsistent in studies.

FMT has been applied in experimental dry eye syndrome. SS dry eye and non-SS dry eye human-delivered FMT transplanted into germ-free C57BL/6J female mice resulted in decreased corneal barrier integrity and decreased concentrations of CD45^+^ CD4^+^ FOXP3^+^ in cervical lymph node cells, indicating that Treg cells could modulate gut microbiome or vice versa. Surprisingly, the offspring of the FMT SS-transplanted mice had decreased CD4^+^ FOXP3^+^ cells in cervical lymph node tissues and spleens. The results suggested that gut microbiota features could be vertically transferred from parents to the offspring, and may be involved in Treg development in the offspring [59]. In another experimental study, FMT was provided by oral gavage into C57BL/6J female germ-free recipients from healthy mouse donors to determine if gut recolonization could improve the dry eye phenotype. The results indicated similar features of the stool microbiota composition in both recipients and donors. The disruption of the corneal epithelial barrier was minimalized, the density of goblet cells improved and the formation of autoreactive CD4^+^ T cells decreased in the lacrimal gland of recipients. Moreover, transplanted microbiota in experimental desiccating stress model resulted in a 50% increase of conjunctival goblet cells, showing the benefits of gut microbiota [47]. Notably, CD25 knock-out (CD25KO) germ-free mice (spontaneous multi-organ inflammatory disease model) developed SS-like symptoms. Similarly to the previously mentioned experimental studies, a decreased corneal barrier integrity and decreased density of goblet cells were reported. Additionally, the expression of IFN-γ, IL-12 and a higher frequency of pathogenic CD4^+^IFNγ^+^ cells were shown. After the exposure to commensal gut bacteria delivered from FMT of the C57BL/6J mouse healthy donors, CD4^+^IFNγ^+^ cells decreased and the SS-like phenotype improved. The results indicated that the colonization of gut bacteria could impact immunoregulation pathways [48].

To our knowledge, one clinical trial applying FMT in SS intervention was finished in 2020 (NCT03926286) and has been recently published as FMT in individuals with immune-mediated dry eye. A study group of 10 individuals with dry eye symptoms, but also other immune comorbidities, received two enema applications of FMT from healthy donors. Surprisingly, the relative abundance of *Faecalibacterium*, *Prevotella* and *Ruminococcus* was significantly decreased compared with donor fecal samples at baseline. Considering that the FMT fecal transplants originated from healthy donors, these results present a completely opposite information than proposed in the study Wu and colleagues mentioned above. Moreover, the clinical trial showed elevated genera *Alistipes*, *Streptococcus* and *Blautia* in dry eye patients at baseline. After FMT treatment, the gut microbiota of recipients shifted towards the microbial composition of the donor in 80% of the individuals. Unfortunately, the microbiota of the recipients was still significantly different from that of the donors, and no significant changes were recorded before and after the treatment within recipient samples. Despite this, the symptoms of as much as 50% of the study respondents improved 3 months after FMT [28].

Based on the results presented in this chapter, the involvement of bacterial taxa in SS development and progression is rather inconclusive. While studies using FMT treatment were partially successful in improving SS symptoms, this was likely not caused by the changes in the microbial profile of the participants.

## 11. Conclusions

Gut microbiota dysbiosis has been reported in a number of extraintestinal diseases, including cardiovascular or neurological disorders. The spectrum of pathological states with a proposed role of gut microbiota further expands. However, it is questionable as to what extent are the changes in gut microbiota composition the cause or the consequence of the disease. Autoimmunity recently emerged as a possible outcome of the interplay between the gut dysbiosis and genetic setting of the patient. Several authors declared that autoimmunity starts or is sustained in the gut.

FMT has been successfully used as a therapeutic tool in gastrointestinal pathologies, such as *Clostridioides difficile* infection or inflammatory bowel disease. Recent research showed its therapeutic potential in several autoimmune diseases. Most of the knowledge comes from experimental studies using mouse recipients of the fecal transplant, and only a small number of clinical studies investigating the therapeutic effect of FMT in autoimmunity have been published (Table 1, Table 2, Table 3 and Table 4). However, the positive results of these few clinical studies warrant further exploring of the role of dysbiosis in the pathogenesis of autoimmune diseases. We hypothesize that maintaining or restoring the eubiosis will likely become a part of clinical guidelines for the effective management of selected autoimmune diseases, which can increase the effect of standard pharmacologic therapy. FMT will possibly represent a common tool to achieve this goal. The results of the ongoing clinical studies and experimental research in animal models will shed more light on the potential of FMT in autoimmunity in the upcoming years.

Despite having appeared for the first time almost two millennia ago, FMT has not been considered as a viable therapeutical approach until recently [15]. Currently, research is ongoing to prove its safety as well as usefulness in the treatment of diseases of the gastrointestinal tract. However, as our collective understanding of the intricate links and connections between microbial balance and the immune system grows, so does the range of FMT applications. In this manner, it is already becoming apparent that the usefulness of FMT reaches beyond the alimentary canal into various organs and body structures which were previously not appreciated as likely FMT targets [140].

While the therapeutic potential of FMT is far from being adequately explored, FMT is likely to become increasingly recognized as a valuable tool. For instance, FMT-driven microbial rebalance may act as a response to the ever-growing threat of bacterial antibiotic resistance. Although FMT is currently viewed more as a therapeutical approach, it may become of great importance in deciphering the complicated microbial relationships occurring in the gut both before and during inflammation. In this aspect, studies focused on bacterium-bacterium and bacterium-cell communication, undoubtedly one of the deciding factors shaping the actions of the immune system, may yield advances in the understanding of the intestinal microenvironment. Finally, FMT may help to explain and uncover new connections in the gut-brain axis which trigger and/or contribute to neurological diseases, including MS or even depression [141].

## Figures and Tables

**Table 1:** Published clinical studies applying FMT in autoimmune diseases. The table summarizes recently published clinical studies in which human donor stool was applied to ameliorate the onset, slowdown the progression or suppress the symptoms of autoimmune diseases, namely celiac disease, multiple sclerosis, psoriatic arthritis, rheumatoid arthritis, Sjogren’s syndrome and type I diabetes. The table summarizes the main outcomes of FMT application and highlights interesting outcome results.

Disorder	Main Outcome	Outcome Details	References
Celiac disease	Successful	Cured *Clostridioides difficile* infection. Mitigation of symptoms of celiac disease.	[25]
Multiple sclerosis	Potential but more research is necessary	A donor-specific alteration of gut microbiota. No statistically significant changes of pro-inflammatory regulatory cytokines.	[13]
Multiple sclerosis	Potential but more research is necessary	An increase in short chain fatty acids (SCFA) genomic pathways post-FMT. A positive correlation between the abundance of microbial SCFA pathway gene content and serum brain-derived neurotrophic factor. Species *Faecalibacterium prausnitzii* elevated. Butyrate, propionate, total SCFA and total-butyrate-to-total SCFA ratio concentrations increased in 2 out of 5 post-FMT measurements.	[26]
Psoriatic arthritis	Potential but more research is necessary	Acceptable and safe FMT application. No life-threatening effects.	[12]
Psoriatic arthritis	Failed	Health assessment questionnaire disability index improved more in the placebo group compared with the FMT group.	[27]
Rheumatoid arthritis	Successful	Successfully cured with FMT. A decrease in rheumatoid factor, disease activity score-28 and improvement of the health assessment questionnaire Disability Index.	[11]
Sjogren’s syndrome	Failed	A donor-specific alterations of gut microbiota. However, microbiota of recipients still significantly different from donors. No significant changes before and after the treatment within recipient samples. Despite this, improved symptoms in 50% of study respondents.	[28]
Type 1 diabetes	Potential but more research is necessary	Type I diabetes progression slowed down. Stimulated C peptide levels were preserved in the autologous FMT group compared with healthy donor FMT group. Small intestinal *Prevotella* was inversely related to residual beta cell function.	[29]

**Table 2:** Ongoing clinical trials applying FMT as an intervention in autoimmune diseases. The table summarizes ongoing clinical trials in which human donor stool was applied to ameliorate the onset, slowdown the progression or suppress the symptoms of autoimmune diseases, namely celiac disease, multiple sclerosis, psoriatic arthritis, rheumatoid arthritis, Sjogren’s syndrome and type I diabetes. The table summarizes the current status of the study as well as the specific identifier and full name of the study.

Disorder	Current Status	Identifier and Full Name of the Study	References
Multiple sclerosis	Completed	NCT03975413 Single-arm, non-randomized, time series, single-subject study: fecal microbiota transplantation (FMT) in multiple sclerosis	[30]
Multiple sclerosis	Active, not recruiting	NCT03594487 Fecal microbiota transplantation (FMT) of FMP30 in relapsing-remitting multiple sclerosis: a phase 1b clinical trial to evaluate feasibility, safety, tolerability and effects on immune function	[31]
Multiple sclerosis	Recruiting	NCT04203017 Allogeneic fecal microbiota transplantation as a consolidation treatment after autologous hematopoietic stem cell transplantation in patients with multiple sclerosis	[32]
Multiple sclerosis	Terminated (primary investigator passed away)	NCT03183869 Fecal microbial transplantation in relapsing multiple sclerosis patients	[33]
Multiple sclerosis	Recruiting	NCT04096443 A pilot study of oral FMT (fecal microbial transplant) in subjects with multiple sclerosis	[34]
Multiple sclerosis	Not yet recruiting	NCT04150549 Fecal microbial transplantation for relapsing multiple sclerosis patients—a placebo-controlled, double-blinded, randomized trial	[35]
Multiple sclerosis, Psoriatic arthritis, Celiac disease	Recruiting	NCT04014413 Safety and efficacy of fecal microbiota transplantation: a pilot study	[36]
Psoriatic arthritis	Completed	NCT03058900 Efficacy and safety of fecal microbiota transplantation (FMT) in patients with peripheral psoriatic arthritis: a 6-month, double-blind, randomized, placebo-controlled trial	[37]
Rheumatoid arthritis and Psoriatic arthritis	Unknown	NCT03944096 Efficacy and safety of fecal microbiota transplantation in patients with rheumatoid arthritis refractory to methotrexate: a 24-week, double-blind, randomized trial	[38]
Rheumatoid arthritis, Psoriatic arthritis	Not yet recruiting	NCT04924270 Safety and clinical efficacy associated with fecal microbiota transplantation performed in treatment-naïve patients with newly diagnosed rheumatoid arthritis, reactive arthritis, ankylosing spondylitis, psoriatic arthritis, gouty arthritis, psoriasis, hidradenitis suppurativa, pulmonary sarcoidosis, Crohn’s disease and ulcerative colitis: a 52-week, double-blind, randomized, placebo-controlled, exploratory trial	[39]
Sjogren’s syndrome	Completed	NCT03926286 Fecal microbial transplant for Sjogrens syndrome	[40]
Type 1 diabetes	Recruiting	NCT04749030 Fecal microbiota transplantation for patients with diabetes mellitus type 1 and severe gastrointestinal neuropathy: a randomized, double-blinded safety and pilot-efficacy study	[41]
Type 1 diabetes	Recruiting	NCT05323162 Encapsulated fecal microbiota transplantation to preserve residual beta cell function in patients with recently diagnosed type 1 diabetes mellitus	[42]
Type 1 diabetes	Unknown	NCT04124211 Fecal microbiome transplantation (FMT) for type 1 diabetes	[43]

**Table 3:** Therapeutic experimental studies applying FMT in autoimmune diseases. The table summarizes experimental studies in which animal donor stool was applied to ameliorate the onset, slowdown the progression or suppress the symptoms of autoimmune diseases in animal models, namely multiple sclerosis, rheumatoid arthritis, Sjogren’s syndrome and systemic lupus erythematosus. The table summarizes the main outcome of FMT application and highlights interesting outcome results.

Disorder	Main Outcome	Outcome Details	References
Multiple sclerosis	Successful	A reduction of cord pathology and ameliorated disease severity.	[44]
Multiple sclerosis	Successful	The onset and amelioration of the disease slowed down post-FMT. A decrease of 13 bacterial taxa, an increase of 17 taxa. Numerous bacteria-specific negative and positive correlations described. Reduced clinical scores and cumulative disease scores. Increased expression of a tight junction protein. Decreased expression of neurofilament light chain protein.	[45]
Rheumatoid arthritis	Successful	A positive effect of tuna elastin peptides transmitted by FMT.	[46]
Sjogren’s syndrome	Successful	A donor-specific alteration of gut microbiota. Minimalized disruption of the corneal epithelial barrier. Improved density of the goblet cells. Improved autoreactive CD4^+^ T cells. Increase of conjunctival goblet cells.	[47]
Sjogren’s syndrome	Successful	Improved corneal barrier function and Sjogren’s syndrome-like phenotype. A decrease of CD4^+^IFNγ^+^ cells.	[48]
Systemic lupus erythematosus	Successful	Alleviated systemic lupus erythematosus symptoms after prednisone-regulated gut microbiota FMT. Decreased relative abundance of genera *Ruminococcus* and *Alistipes*. Retained relative abundance of *Lactobacillus*.	[49]
Systemic lupus erythematosus	Successful	Alleviated gut dysbiosis caused by prior antibiotics administration and suppressed SLE progression.	[50]

**Table 4:** Pathologic state-inducing experimental studies applying FMT in autoimmune diseases. The table summarizes experimental studies in which human or animal donor stool was applied to induce the pathologic states of autoimmune diseases in animal models, namely Grave’s disease, Hashimoto´s disease, rheumatoid arthritis, Sjogren’s syndrome, type I diabetes and systemic lupus erythematosus. The table summarizes the main outcomes of the FMT applications and highlights interesting outcome results. Transplantation from human donor into animal model recipient (H-to-A). Transplantation from animal donor into animal model recipient (A-to-A).

Disorder	FMT Transfer	Main Outcome	Outcome Details	References
Graves’ disease	A-to-A	Successful	Gut microbiota alternations post-FMT. A greater decrease of T3 and T4 hormone concentrations, increased liver expression of type 2 deiodinase and better recovery of hypothyroid-induced resting metabolic rate back to normal.	[51]
Graves’ disease	H-to-A	Successful	A donor-specific alteration of gut microbiota. Increased disease severity, reduction in Shannon diversity, increased richness indices. Decreased abundance of *Bacteroides* compared with control mice.	[52]
Graves’ disease	H-to-A	Successful	Increased incidence of Graves’ disease. An increase of serum total thyroxine concentrations, thyroglobulin antibodies and IL-17A. Decreased serum concentrations of IL-10.	[53]
Hashimoto’s disease	H-to-A	Successful	A decrease of serum total thyroxine concentrations, mRNA expression of occludin, junctional adhesion molecule-A and zonula occludens-3211.	[54]
Rheumatoid arthritis	A-to-A	Successful	Physical changes, e.g., cartilage alterations, paw deformities present. Increased concentration of tissue inflammatory mediators. Activation of CD4/CD8^+^ T-lymphocytes. Behavioral modifications. Occult bleeding with gut tissue disruption.	[55]
Rheumatoid arthritis	H-to-A	Successful	Depression-like phenotypes. Alterations of gut microbiota composition. Increased percentage of CD3e^+^ and CD4^+^ T-lymphocytes in Peyer’s plaques and spleen. Increased Th1/Th2 index and decreased CD25^+^ and FOX3^+^ Treg cells. Downregulation of synaptic proteins. Negative correlation of *Bacteroides*, *Phascolarctobacterium* with the Th1/Th2 index and positive correlations with a decreased percentage of Treg cells in Peyer’s plaques and spleen. Twelve promising rheumatoid bacterial biomarkers proposed.	[56]
Rheumatoid arthritis	A-to-A	Successful	Severe joint swelling. Maximum arthritis score observed in FMT transplanted mice compared to non-FMT mice.	[57]
Rheumatoid arthritis	A-to-A	Failed	Attenuation of experimental arthritis more efficient without antibiotic treatment and FMT administration.	[58]
Sjogren’s syndrome	H-to-A	Potential but more research is necessary	Decreased corneal epithelial barrier integrity and decreased concentrations of CD45^+^, CD4^+^, FOXP3^+^ in cervical lymph nodes cells. Decreased CD4^+^, FOXP3^+^ cells in cervical lymph nodes tissue and spleen in offspring of FMT-transplanted mice.	[59]
Systemic lupus erythematosus	A-to-A	Successful	Changes in immune cell distribution in recipients. Upregulated expression of lupus susceptibility genes.	[60]
Systemic lupus erythematosus	H-to-A	Successful	A lupus-like phenotypic features in FMT transplanted mice. Increased serum autoimmune antibodies, imbalanced cytokines, altered distribution of immune cells in mucosal and peripheral immune response and upregulated expression of genes related to systemic lupus erythematosus. Metabolism of histidine modified.	[61]
Type 1 diabetes	H-to-A	Potential but more research is necessary	Delayed onset of Type I diabetes. The pace of beta cell loss not transferable to the mouse model.	[62]

## Data Availability

Not applicable.
